# One Solution to the Arsenic Problem: A Return to Surface (Improved Dug) Wells

**Published:** 2006-09

**Authors:** Sakila Afroz Joya, Golam Mostofa, Jabed Yousuf, Ariful Islam, Altab Elahi, Golam Mahiuddin, Mahmuder Rahman, Quazi Quamruzzaman, Richard Wilson

**Affiliations:** ^1^ Dhaka Community Hospital, 190/1 Baro Moghbazar, Wireless Railgate, Dhaka 1217, Bangladesh; ^2^ Department of Physics, Harvard University, Cambridge, MA 02138, USA

**Keywords:** Arsenic, Sanitation, Water supply, Coliform, Bangladesh

## Abstract

Arsenic contamination in drinking-water in Bangladesh is a major catastrophe, the consequences of which exceed most other man-made disasters. The national policy encourages the use of surface water as much as possible without encountering the problems of sanitation that led to the use of groundwater in the first place. This paper describes the success of the Dhaka Community Hospital (DCH) team and the procedure in implementing sanitary, arsenic-free, dugwells. The capital cost for running water is US$ 5–6 per person. Sixty-six sanitary dugwells were installed in phases between 2000 and 2004 in Pabna district of Bangladesh where there was a great need of safe water because, in some villages, 90% of tubewells were highly contaminated with arsenic. In total, 1,549 families now have access to safe arsenic-free dugwell water. Some of them have a water-pipe up to their kitchen. All of these were implemented with active participation of community members. They also pay for water-use and are themselves responsible for the maintenance and water quality. The DCH helped the community with installation and maintenance protocol and also with monitoring water quality. The bacteria levels are low but not always zero, and studies are in progress to reduce bacteria by chlorination.

## INTRODUCTION

The arsenic problem in Bangladesh has been widely discussed. Beginning about 30 years ago, people in Bangladesh have been abstracting groundwater by sinking tubewells. The wells were cheap, and water seemed to be free of bacteria that cause cholera. Although this seemed like a miraculous solution to the nation's drinking-water problems, it produced its own very serious problems. About 30% of wells contained too much arsenic. The Government of Bangladesh was alerted to the ailments caused by arsenic as early as 1993, and physicians at the Dhaka Community Hospital (DCH) saw many victims in 1996. The ailments were not brought to the world's attention until the first (of eight) International Conference on Arsenic, held jointly by the DCH and Jadavpur University, Kolkata, West Bengal, India, in February 1998 (1). At the same time, the DCH, under a contract from the Ministry of Health and Family Welfare, Bangladesh, and the United Nations Development Programme, rapidly assessed 500 highly-contaminated villages (2). The DCH and Jadavpur University also carried out detailed surveys in many other villages (3). At that time, several ‘obvious’ conclusions were as follows: (a) a short-term solution might be acceptable if it was implemented on a wide scale at once; (b) a long-term sustainable and affordable solution should fit into a national water policy; (c) there is no reason for delay; short-term solutions should be implemented at once; and (d) a simple return to unsanitary surface waters is undesirable.

The proposals made immediately were to: (a) have a national survey of wells; (b) encourage switching of all the wells (use of a well without arsenic); (c) install temporary (household scale) arsenic-removal devices; and (d) use deep wells (deep enough to penetrate a clay layer). The implementation of these proposals has been slow and, seven years later, the short-term plan became long-term. As a consequence, many villages were still without any pure drinking-water. Switching of wells has been variable: some estimates are that only 30% of villagers switched wells. Scientists at the Columbia University found that the percentage was 60 in the area they studied, perhaps because they had an intense village-education programme (19). The arsenic-removal devices proved too hard for many villagers to use, and many of them were unsatisfactory and were, thus, abandoned (4).

Some scientists have cautioned against indiscriminate use of deep wells. Although arsenic contamination in deep layer is at present much smaller than arsenic contamination in ordinary tubewells at a depth of 40 meters, it is unclear whether it will always remain so (5). In Dhaka, continuous extraction of groundwater is non-rechargeable at the same rate of extraction. According to a report of the Water and Sewerage Authority (6), the underground water level of Dhaka city is going down continuously due to extraction of water.

In 2003, the Government of Bangladesh adopted a national water policy (7), giving a priority to the use of surface water among other options. These surface-water options included: (a) encouraging a return to surface (dug) wells, but with strict adherence to the sanitary standards of the World Health Organization (WHO), (b) use of sand-filters to filter pond water or river water, and (c) storage of rainwater.

In all solutions, involvement of the local community is essential. The DCH is particularly suited to pilot projects at the local community level because each of their 40 local clinics can act as a focus for action. The Hospital chose the first of these surface-water solutions—use of dugwells—for the first demonstration facility in Pabna district. This report describes three phases of the work starting in 2000 until 2003, while also exploring an indication of further developments in another district since 2003. So far, the groups that have been actively studying and installing deep tubewells have been successful and have brought pure water to over a million people. However, there may be locations where deep wells are not suitable, and their widespread use may perhaps be undesirable. For these reasons, we believe that all solutions should be studied, and we make no premature claim on whether, and/or where, a particular solution will prove to be the best.

## THE DCH DUGWELL DEMONSTRATION (PILOT) PROJECT

Dugwells were used for a long time in Bangladesh, but were replaced by tubewells due to their simplici-ty and the absence of bacterial contamination without the apparent need of careful maintenance. A return to dugwells, therefore, seems to be an obvious possibility. However, this has not been uniformly successful. This project demonstrated that it is possible to have bacteria-free wells if due care is taken and if, in particular, requirements of the WHO were followed (8). While this is obvious in a temperate climate, such as the UK, it is far from obvious in the village conditions of Bangladesh. There were, therefore, several issues to be explored: Will the wells be free of bacteria? Will the wells be free of arsenic and other undesirable chemicals? What will be the cost? What maintenance is necessary? Are there other conditions, such as limited choice of sites, that are necessary to achieve these aims? Will the wells be acceptable to the people?

After the start of the project, the DCH noted that the electrification programme of the Government of Bangladesh had already brought electricity to 50% of all villages and had the aim of bringing electricity to them all by 2020. Electricity makes it easy to install an electric pump to raise water to a storage tank, from which it is gravity fed by pipeline to a number (6 or more) of individual houses. This has proved to be very popular and is a major step towards the widespread acceptability of this solution. Ahmad *et al.* found through a survey that the availability of running water is more important in public perception than the fact that water is arsenic-free (9). The Bangladesh Arsenic Mitigation Water and Supply Project (BAMWSP) has also stated its intention of providing 30 pipeline systems (10), but we have no further information about these.

Although the project began in late 1999, it started properly by April 2002. In the first phase, 39 wells were dug (or in some cases reconditioned) by February 2003. These wells supplied water to 631 families and served 3,250 users. Only one had a pipeline system attached. In phase 2, 17 new wells were dug, and all had pipeline systems installed. Water was supplied to another 518 families, and 2,903 users were served. In phase 3, nine old wells were renovated (brought up to sanitary standards of WHO), and one new well was dug; all with electric pump, storage tank, and pipeline. This supplied water to another 400 families with 2,400 users.

**Fig. 1.**
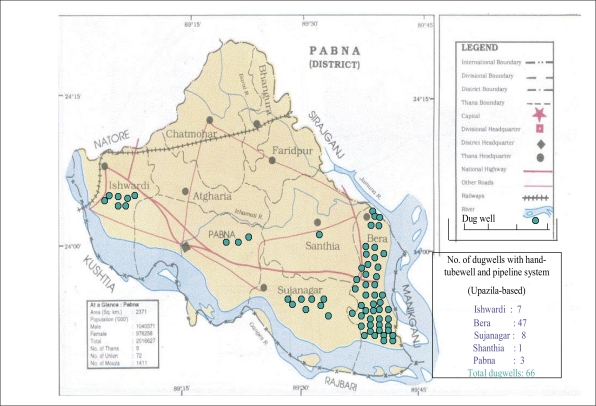
Location map of dugwells (Wilson project)

In total, 66 sanitary dugwells were installed during this demonstration pilot project in the Pabna region. This region was chosen for a number of reasons. First, there seemed to be a great need in this area as nearly all tubewells in several villages showed excessive levels of arsenic. In several villages, patients with evidence of arsenic-related lesions were found. Second, the DCH has a clinic in Pabna where patients may be seen and where water samples were analyzed. Third, epidemiological studies of arsenic lesions are being studied in this region by the DCH, together with a group from the Harvard University. The general geographical location of these wells is shown in Figure 1.

### Procedure for installation of a dugwell with pipeline

March and April, which are the driest months in the country, are the best times to dig a well. During this period, groundwater is at its lowest level meaning that if the well hits water at this time it will always hit water. The community owns the wells and is responsible for their installation and maintenance. The DCH does not own the wells, but merely facilitates, and this paper reports on these. Because of the importance of full participation by the community and the fact that this has not always been achieved, we outline the procedures the DCH has adopted to ensure this responsibility.

The DCH found that there were several major distinct activities which could not be omitted if success was to be achieved, including: community mobilization, committee formation, training of community workers and caretaker, and site selection,

### Water-supply network

**Fig. 2.**
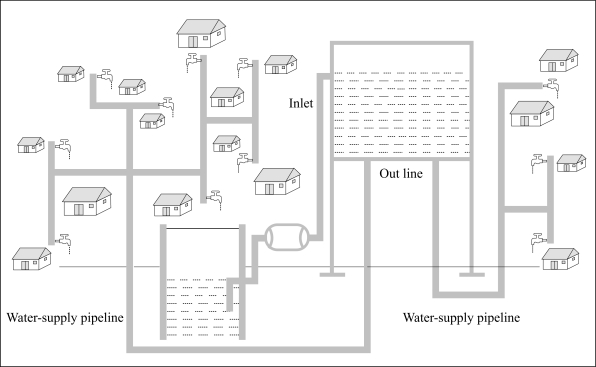
Water-supply network

Water from (large) dugwell or from the river sand-filter is pumped up to a overhead tank and this supplied to various households or to some places arranged by the community for easy collection as shown in Figure 2.

### Community meeting for motivation, community participation, and monitoring of water quality

#### Community mobilization by community meeting

Various mobilization and motivational activities, such as courtyard meetings, were conducted to increase public awareness. Several meetings with the community were held in each village. Along with DCH personnel, influential local people and elected representatives from the Ministry of Local Government, Rural Development & Co-operatives, Government of Bangladesh, attended the meetings. The community people, including women, the poor, and arsenic patients shared their situation, needs, opinions, and preferences about mitigation options with the DCH and others.

#### Committee formation

In each village, a committee was formed to supervise the implementation of each stage. Each committee was responsible for maintaining the surface-water option provided to them. The DCH and the committees worked together to plan installation and maintenance of the option. The committee accepted responsibility to collect community contributions and decided prices for the use of water for each family. A caretaker collected money from users of water (usually 20 taka or 35 cents a month for each family). Each family was provided with a water card for payment.

#### Training

Local mistris were selected for the construction and maintenance of the options. They were trained on construction-work options by the DCH trainers who also trained caretakers and users of options.

#### Site selection

Sites for wells were selected in areas highly contaminated with arsenic. This was done after consultation with the community. Preference was given to locating the wells near patients' families and the poor. All 66 sites satisfied the guidelines provided for site selection, including but not limited to: (a) preparing a dugwell 30–40 feet away from latrine and dumping ground of waste materials; (b) animals are penned away from dugwell; and (c) the dugwell is installed at a safe distance from cropland and industrial areas, etc.

A detailed check-list for adequacy of the site selection is being prepared.

#### Installation

A hole is dug with a diameter of about 36 inches. The depth of the well varies from place to place. A ring of cement or baked clay is set from bottom to top, and the rings are joined (sealed) by cement to keep well-water safe from contaminated surface water. An apron of about four feet is made around the head wall and a 30–40-feet drain is constructed at the ground level to avoid water seeping into the well around the head wall. An electric pump pumps water from the dugwell to an overhead reservoir with a capacity of 3,000 litres. This overhead tank is installed on an iron stand, which is 15-feet tall. The stand is fixed on the ground with RCC work. A main water-supply pipe (made of 3/4″ plastic) is connected with the tank for the distribution of water at the household level. A pipeline of ½-inch plastic is connected with the main line to supply water to each individual household. Forty to fifty households are connected with a single main supply line. To prevent accidents during construction of dugwell, such as side-soil collapsing and occasional asphyxiation from carbon dioxide and methane gases, rope, ladder, a Bosun's chair, and other safety equipment are kept at the site. A 30–40-feet drain is constructed at the ground level to avoid water seeping into the well around the head wall.

#### Monitoring of water quality and importance of measurements

One of the most important functions of the village committee is to continually monitor and guarantee the quality of water in accordance with the WHO guidelines and with the quality guidelines prepared by the DCH in consultation with experts. The village committee can call upon the advice and help of DCH and others. To successfully carry out these functions, this aspect of implementation is so important and so often neglected, but was neglected in some cases as noted below, that we emphasize it further in a separate section below.

Failure to make adequate measurements has been at the heart of the dire arsenic problem in Bangladesh. For 20 years, no-one measured the arsenic levels even in a small sample of the millions of tubewells until it was too late. More recently, many small-scale arsenic-removal devices were installed without adequate measurements to demonstrate their efficacy. Some NGOs returned to surface waters without following the sanitary guidelines of WHO and without measuring possible bacteriological contamination. For this and other reasons, the DCH has insisted on measurements from the outset and has recommend that a copy of all measurements be made publicly available. It is important that not only the individual who has the well be convinced of accurate measurements but also the DCH as a whole and through the wider community. The measurements of this pilot project are available in the Appendix (more details are at http://DCHtests.arsenic.ws).

### Measurement of arsenic concentrations

It is extremely unlikely that aerated surface waters will have the same level of arsenic that the deeper wells do. One present theory is that arsenic is dissolved by water when there is an anoxic environment and, therefore, having a well open to air is helpful. There have been no reports of chronic arsenic poisoning in thousands of years of dugwell-use before tubewells came into use. Dipenkar Chakraborti reported on the measurement of 700 dugwells in Bangladesh and West Bengal and found that 90% of the tubewells had levels of arsenic less than 30 ppb, and only a few had 50 ppb (11). More recently, in phase 1 of the “Risk Assessment of Arsenic Mitigation Options (RAAMO)”, the Arsenic Policy Support Unit (APSU) found that 1% of dugwells they surveyed had arsenic above 50 ppb but none had the very high levels found in ordinary tubewells (12). This suggests that the frequency of arsenic measurement is less important than measurements of coliform bacteria. The problem with the requirements for measurement of arsenic is that we are asking to reliably measure levels of arsenic at 50 ppb in water when other chemicals are present at much higher levels. Laboratory instruments can, in principle, achieve this with no difficulty by gas chromatography (at a cost of $30,000 for each piece of equipment). Moreover, this involves taking samples in the field and bringing them back for measurement. The large cost of laboratory instruments makes accurate measurement difficult and inaccessible.

A simple calculation performed four years ago showed that there was barely enough equipment in Bangladesh to measure each well every 1,000 years! Worse still, an unpublished draft report by the International Atomic Energy Agency (IAEA) showed some alarming disagreements between measurements in different laboratories in an inter-laboratory comparison (14). Measurements in the field were even worse as they depended upon the training of personnel. A group of scientists from Bangladesh and West Bengal reported on their comparisons of measurements of arsenic concentrations in 2002 and insisted that “facts and figures demand improved environmentally friendly laboratory techniques to produce reliable data” (14). However, despite the challenges, there is hope on the horizon. More recent (2005) laboratory comparisons of water samples with the IAEA laboratory showed that several laboratories were in full agreement regarding measurements. Some laboratories failed in the precision of their measurements. The lower precision (25%) is not important for the present purpose because all the measurements of arsenic here reported are only upper limits. Scientists from the Columbia University found that the Hach-kit can be reliable if used in a slightly different manner than recommended by the manufacturer (15), and the BAMWSP switched to this kit but this information was not available to the DCH at the time.

It is not anticipated that levels of arsenic will be high in surface wells, whether tubewells or dugwells, and it has been suggested that aerated dugwells have even less arsenic. While measurement of arsenic is important, it is less important than for measurement of coliform bacteria. For measurements mentioned in the Appendix, the silver diethyldithiocarbamate method was used. It is well-known that this method is difficult to use <5 ppb. Cross calibrations with measurements at the Harvard University (but not using these exact samples) using gas chromatography showed the measurements to be unreliable <5 ppb. For this reason, only an upper limit is quoted in the table in the Appendix.

### Measurement of coliform bacteria

Although the measurement of coliform bacteria is, in principle, much simpler than measurement of arsenic concentrations, the reliability in practice is critically important, and the frequency of measurements needed for dugwells is greater than the required frequency of measurements of arsenic. Many users of dugwells have found considerable amounts of coliform bacteria. There is a general agreement that the measurement of coliform bacteria can be reliable. For first measurements, the DCH had no equipment of its own. Measurements by other institutions were expensive and unreliable and are not reported here. In 2001, we acquired a ‘Delagua’-kit (16), designed at the University of Surrey, and used it for all measurements for the 66 dugwells. More recently, the coliform vial from a Jal-Tara measurement-kit (17) from Clean India in New Delhi has been used for giving an initial qualitative test to determine whether a full measurement is necessary. The initial measurements of the 66 dugwells in this project are shown in the Appendix. They were repeated by the DCH every three months for a little over a year before handing over the measurements to the community managers. Results of the initial set of 5 or 6 tests is available on the web (http://DCHtests/arsenic.ws) and showed low levels of total coliform and zero of faecal coliform. In retrospect, it seems probable that these measurements were immediately after maintenance: cleaning and disinfection with lime, and may not be a good indication of behaviour after a few weeks.

In 2004, questions were raised about the quality of DCH wells. Other organizations had installed dugwells with less apparent success. Although the installation of many, if not most, of these wells had not followed the WHO guidelines, some had and showed high levels of bacteria. A report from the APSU measured median (mean) levels of total thermoluminescent coliform (TTC) of 47 (163) per 100 mL in the dry season and 820 (1,998) per 100 mL in the wet season in a sample of 36 dugwells of all types (apparently including those not following the WHO criteria) but not including any wells dug by the DCH (12). This large difference between the median and the mean suggests that the distribution is skewed. Although not stated, despite our questions, this is probably due to a few wells with high levels, presumably the old uncovered wells. This makes the report less useful for public-policy purposes. However, this naturally led to suspicion of the wells dug by the DCH.

Accordingly, 20 of the 66 wells were tested again on a frequent basis for a year (July 2004–June 2005). A different coliform-measuring equipment was used from MacConkey, because the culture medium—MacConkey (purple) broth—is more readily available in Bangladesh than the culture medium for the Delagua-kit (membrane lauryl sulphate broth). This time, tests were only made for faecal coliform and not for total coliform. The measurements revealed problems, of which we were not previously aware. It can be seen from a plot for six of these wells in Figure 2 that the levels of coliform bacteria were high in July 2004, at the start of the monsoon, and were, therefore, in violation of the present standard, but soon dropped to less than 10 structures per 100 mL during the monsoon when they would be expected to rise (although one rose again). It is unclear why these results were obtained. This was the first time that the ‘multiple tube’ method was used, and the first measurement might have been an error. It is also possible that the guardians of the wells had not applied lime when appropriate during the previous 6–12 months.

These faecal coliforms are not dangerous in themselves but indicate that water is contaminated with human or animal wastes and may contain dangerous pathogens. In principle, both U.S. Environmental Protection Agency and WHO state that there shall be no faecal coliform (18). These wells were all in a region where a nearby DCH clinic exists, and no unusual health effects have been reported. This may be due to a resistance to infection of Bangladeshis after childhood, or regular water boiling (which is not done nation-wide!). Nonetheless, it is highly desirable to keep the bacteria levels low.

**Fig. 3.**
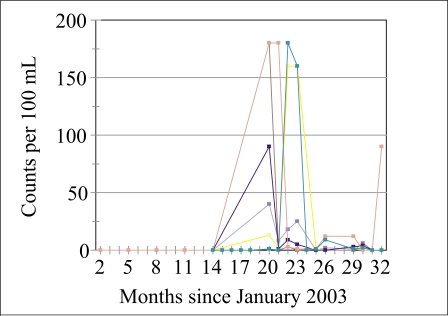
Faecal coliform bacteria

Unfortunately, the dates when maintenance was performed were not recorded for the data in Figure 3. Research continued, with careful recording of all relevant features of the wells, to understand the reasons for the high levels of coliform when they occur and to understand the required frequency of monitoring. In addition, the DCH is following the suggestions by many sanitation experts and recommending that the wells be chlorinated regularly to extend the period of safe use. This procedure is adopted in much of the world and has been successful in neighbouring West Bengal, but has not been widely adopted in Bangladesh. Implementation is underway.

The capital cost of the wells was approximately US$ 70,000, which does not include the cost of DCH planning and supervision. The capital cost is falling with time as we learn how to use indigenous materials and local labour. Detailed breakdown of the cost for different types of installation is shown in Table 1.

**Table 1.** Typical costs of different types of dugwells, excluding DCH supervision

Type of dugwells	Tap points (average)	Families covered approx.	Population covered approx.	Total cost		Per-person cost	
				Tk	US$	Tk Cl.6/Cl5	US$ Cl.7/Cl.5
Improved new dugwell + pipeline network + water tank (steel) + water tower (steel column)	8	50	250	83,260	1,388	333	5.5
Improved new dugwell + pipeline network + water tank (steel) + water tower (brick column)	8	50	250	92,760	1,544	371	6.2
Improved new dugwell + pipeline network + water tank (steel) + water tower (RCC column)	8	50	250	98,610	1,643	394	6.5
Improved new dugwell + hand tubewell	1	15	75	58,754	979	783	13.0
Recondition dugwell + pipeline network + water tank (steel) + water tower (steel column)	8	50	250	50,350	839	201	3.3
Recondition dugwell + pipeline network + water tank (steel) + water tower (brick column)	8	50	250	72,070	1,201	288	4.8
Recondition dugwell + pipeline network + water tank (steel) + water tower (RCC column)	8	50	250	70,220	1,170	280	4.6
Recondition dugwell + hand tubewell	1	15	75	31,394	523	419	7.0

Approx.=Approximately;

DCH=Dhaka Community Hospital;

US$ 1=Tk 60

### Measurement of manganese and other chemicals

The first measurements in the Appendix were of those pollutants that were easily measured with the Delagua-kit. Recently, it has been suggested that manganese is a serious problem in many surface waters and has effects on health that can be as serious as those of arsenic. In response to this suggestion, the Bangladesh University of Engineering and Technology made a search for manganese upon our request. The WHO standard is 0.4 mg/L, and most measurements were below 0.1 mg/L. In two wells at one time only, the measurement was up to 0.6 mg/L. Details of the measurements are available in the website (20).

### Acceptability

The APSU performed a qualitative ‘social assessment’ survey on the acceptability of dugwells they tested (12). Although 79% of persons surveyed stated that the dugwells were acceptable, no specific surveys have been undertaken to provide a quantitative level of acceptability in this study. However, on various visits to the villages by one or more of the authors subsequent to installation, uniform enthusiasm has been observed. People from neighbouring villages have requested the help of DCH, and enthusiasm has particularly been shown for the distribution of water by pipeline because it reduces the distance to fetch water. This was not an issue or question in the APSU study (12). Since the addition to the cost is modest, and it enables more people to be served by the same facility, we encourage that this improvement be undertaken at the same time. We suggest that any further discussion and verification of acceptability should be accompanied by a ‘willingness to pay’ for the improvements.

**Fig. 4.**
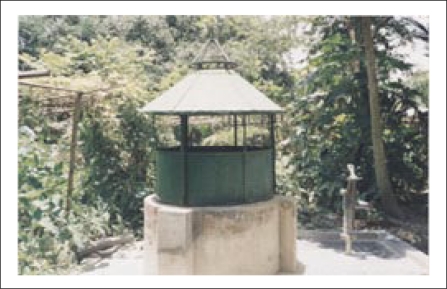
A typical dugwell with an attached tubewell

**Fig. 5.**
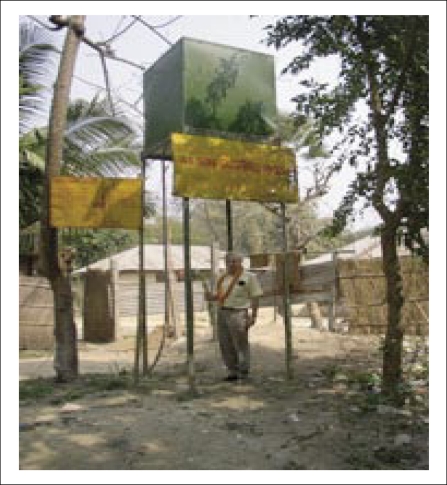
Water-tank from which gravity feeds the houses

**Fig. 6.**
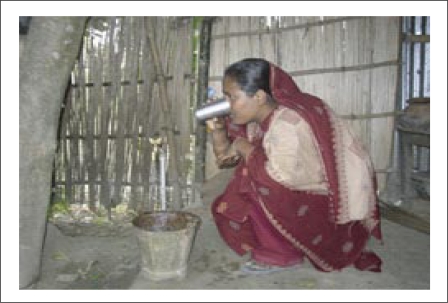
A tap with pure water in a kitchen

Figures 4, 5, and 6 are photographs taken on the well number DWP40 in Mallithapa, Ruppur, Pabna, in 2004, which show respectively a typical dugwell with attached tubewell, the water tank from which gravity feeds the houses, and a tap with pure water in her kitchen for the first time in the history of Bangladesh.

### Subsequent maintenance

The underlying principle of the DCH's assistance to the villages is that the villages own wells and own the responsibility for their good operation. Originally, the DCH suggested, for maintenance, a careful visual inspection every three months. Frequent measurements have been made on the 66 wells and on subsequent wells dug under the DCH guidance, so that the issues may be fully understood. In the long-term, significantly fewer measurements are likely to be necessary. No problems of arsenic and manganese contamination have been found, and it is unlikely that the levels of arsenic will increase rapidly. Therefore, we suggest that a complete chemical analysis needs not be frequent and can be carried out every two years. However, as noted earlier, bacteriological contamination can be serious and can change fast. The DCH is examining chlorination in an attempt to ensure that all wells are completely bacteria-free for long periods after maintenance.

The DCH is urging the community to call for measurements if and when any of the following events occur: (a) after a visual inspection (to be carried out every three months) of the well casing, or the apron, (b) surrounding the well seems cracked, (c) the water begins to smell foul, and (d) the turbidity increases.

The DCH is preparing a detailed advisory and check-sheet for this maintenance. Since, as noted in Table 2, the maintenance can be an appreciable cost item, this remains an important consideration for further study and examination.

**Table 2.** Maintenance cost (per well)

Material	Quantity	Price (Tk)	Total (Tk)
Labour charge for cleaning	5 persons	400	2,000
Potash	100 g		30
Lime	3 kg	10	30
Repairing and fixing broken, leaking, and other damaged parts			1,000
Total: Tk 3,060 (US$51.00) [US$ 1=Tk 60]			
The annual expense for maintaining a dugwell is modest and is borne by the village community. Typically, families pay Tk 10–20 per month (Tk 120–240 per year) which usually includes Tk 60 for maintenance, a small stipend for the caretaker as chosen by the village Option Management Committee and the electricity bill for the pump			
At the present time, the cost of the measurements is borne by DCH			
Cost of full 15 parameter (including arsenic) tests performed initially and when needed (biannually)	Tk 5,500 (US$ 95)		
Cost of preliminary coliform test by Clean India (Jal-Tara) performed quarterly	US$ 1 each, US$ 4 a year		
Cost of measurement of faecal coliform is when indicated by Jal-Tara or other tests (approximately annually)	Tk 400 (US$ 7)		

### Long-term solution

The DCH has always acknowledged that a choice between acceptable actions should be guided by whether the action leads to a long-term solution. In the long-term, we hope that most people of Bangladesh will have access to publicly-supplied pure running water where concerns about purity are handled centrally. The DCH makes no judgment on whether deep tubewells or surface waters will ultimately be the source of water. We note, however, that dugwells with a piped water supply have traditionally been a step in this direction for many countries. For example, in an English village (Binsey in Oxfordshire) in which one of us lived for several years, an open dugwell was used for centuries with a bucket for collection. In about 1920, this well was covered, and a pump was installed; in 1939, a windmill was installed to pump up water to a tank from which a pipe led to every house and cottage; in 1945, a small petrol engine was installed for use when there was no wind; about 1960, a main water line came within a mile of the village, and it was easy to make a spur connection to the village water-supply network. This, of course, would also apply in Bangladesh if water from a deep well were pumped to an overhead tank for distribution.

## CONCLUSION

The use of a sanitary (dug) well has been shown to be a satisfactory and reliable solution for the provision of adequately bacteria-free and arsenic-free running water in several villages in Bangladesh. The capital cost for providing running water is about $5–6 per person. Crucial steps in achieving arsenic- and bacteria-free water seem to indicate: (a) selecting a site suitable for a dugwell (one in peat is sure to smell!), (b) strict adherence to sanitary standards as discussed, for example, by WHO, and (c) ensuring community participation, ownership, and maintenance of each well. In addition, it is likely that regular chlorination, as is practised in many countries, will be necessary to keep bacteria low while reducing the required frequency of maintenance.

This pilot project has been, and is being, extended considerably in the Pabna region where word-of-mouth communication has created a demand by people from other villages. While the installation has been supervised by the DCH personnel, there is a steady increase in the understanding by the villagers themselves. With the financial assistance of United Nations Children's Fund (UNICEF), the DCH has also supervised, since 2003, the installation of 137 wells (three with pipelines) in Sirajdikhan upazila where the DCH also maintains a clinic. We note that the coliform measured in the DCH wells (data also available on the website at http://phys4.harvard.edu/∼wilson/arsenic/remediation/dugwells/DCHtests/Dugwell_tests_for_UNICEF-1.xls) showed non-zero levels of coliform in many wells but much smaller than in those APSU reported in their Tables 4.1 and 4.2.

While the DCH has so far supervised 224 dugwells, bringing pure water to perhaps 50,000 people, this is still only supplying pure water to 0.1% of the population in Bangladesh who are in need. At all of these dugwells, the same careful procedures are adopted. The original 66 dugwells described in this paper were free of faecal coliform bacteria and were very low on total coliform, but about 18 wells dug later in Sirajdikhan upazila were contaminated after an unusually severe flood. The DCH re-treated these wells, and all of them are now safe, with measurements showing a zero or very low coliform bacteria count. In 50 of these wells, the measurement of bacteria count (using the Delagua-kit) and contamination by other metals have been verified by measurements by ICDDR,B. These are noted in the list of measurements for these wells that are on the arsenic website at: http://phys4.harvard.edu/∼wilson/arsenic/remediation/dugwells/Dugwell_tests_for_UNICEF.xls or a shortcut: http://DCHtests.arsenic.ws.

As the dugwell option is further implemented, it is important to use the indigenous materials and measurement techniques whenever possible. This concept was used in this pilot project. A part of the pilot project was clearly to demonstrate all aspects of a remediation method which includes a measurement of its cost-effectiveness. The resources of DCH are limited measuring that, for the widespread use of dugwells, it will be necessary for other groups to come forward. These groups will need to learn the details of the simple technology and learn to work with and supervise the villages in the same way. Hopefully, some groups will take this next step in the coming year.

In addition to dugwells, the DCH, in collaboration with the Government of Bangladesh, UNICEF, and donor agencies, has started to provide other satisfactory surface and sub-surface water-based alternative options of safe water. These include five river-sand filters, nine pond-sand filters, and 1,122 rainwater harvesting units in the arsenic-affected communities. It is likely that chlorination will be necessary for these systems too. These pilot, demonstration projects will be available for others to follow. The DCH also provides training on arsenicosis and arsenic problems through its Institute of Family Health and provides training for overseas medical personnel, e.g. the Nepalese Health Department.

The care that is necessary for the installation and maintenance of sanitary dugwells is greater than originally recognized. However, these have been installed successfully, have proved popular, and could be installed in many other parts of Bangladesh.

## ACKNOWLEDGEMENTS

The authors acknowledge the help of many people who made this work possible. First and foremost, the OPEC Fund for International Development needs to be acknowledged along with other donors which made the project possible. Clean India of New Delhi, started by Dr. Ashok Khosla, provided reliable test equipment at a low cost.

## Appendix. Dhaka Community Hospital-Pilot Sanitary Dugwell Project-Pabna Region

**Table:**

Department of Environmental Research Laboratory Report														
Data				: Analysis of arsenic and bacteriological tests in water samples										
Method of analysis (arsenic)				: Silver diethyldithiocarbamate method										
Method of analysis (bacteriolocical)		: Oxfam-Delagua-kit method			FC=Faecal coliform per 100 mL; TC=Total coliform per 100 mL									
DW code	Name of caretaker	Address			Date of installation or renovation	Families covered	Ph	As conc.	Cond.	TDS	Turb	FC	TC	
									(ppm)	(us)	(ppm)	(NTU)			Date of first testing
							WHO guideline values 1996							
Village	Union	Thana	6.5–8.5	0.01	850	1,000	5	0	0	
DWH-1	Md. Anowar Master	Maddhapara	UBM-01	TBC-01	1999 Nov	12	7.0	<0.03	234	152	<5	0	1	20.06.01
DWH-2	Ramjan Ali Pramanik	Ruppur	UBR-02	TBC-01	1999 Dec	11	7.5	<0.03	212	138	<5	0	0	20.06.01
DWH-3	Md. Akbar Hossain	Ruppur	UBR-02	TBC-01	1999 Nov	10	7.1	<0.03	241	157	<5	0	0	20.06.01
DWH-4	Md. Tipu Sultan	Kazipara	UBM-01	TBC-01	1999 Dec	12	7.5	<0.03	265	172	<5	0	0	20.06.01
DWH-5	Govt. Idara	Bissanathpur	UBJ-03	TBC-01	1999 Nov	15	7.2	<0.03	354	230	<5	0	_2_	20.06.01
DWH-6	Md. Masum Master	Sinduri	UBJ-03	TBC-01	1999 Dec	14	7.0	<0.03	310	168	<5	0	1	20.06.01
DWH-7	Md. Nasir Khan	Sinduri	UBJ-03	TBC-01	1999 Nov	13	7.1	<0.03	241	157	<5	0	0	20.06.01
DWH-8	Md. Mirja Abdul Halim	Aminpur	UBJ-03	TBC-01	1999 Nov	10	7.2	<0.03	246	160	<5	0	0	20.06.01
DWH-9	Md. Jamal Hossain	Aminpur	UBJ-03	TBC-01	1999 Nov	15	7.0	<0.03	260	152	<5	0	1	20.06.01
DWH-10	Md. Majnu Mia	Aminpur	UBJ-03	TBC-01	1999 Nov	12	7.1	<0.03	212	138	<5	0	1	20.06.01
DWH-11	Md. Hanif Sheikh	Kalikapur	UBJ-03	TBC-01	1999 Nov	15	7.0	<0.03	350	228	<5	0	2	20.06.01
DWH-12	Md. Ali Hossain	Kalikapur	UBJ-03	TBC-01	1999 Nov	14	7.0	<0.03	235	153	<5	0	1	20.06.01
DWH-13	Md. Kader Mondal	Kalikapur	UBJ-03	TBC-01	1999 Nov	10	7.0	<0.03	215	140	<5	0	0	20.06.01
DWH-14	Md. Samsur Hossain	Nayabari	UBJ-03	TBC-01	1999 Nov	14	7.2	<0.03	198	129	<5	0	1	20.06.01
DWH-15	Md. Jabber Master	Nayabari	UBJ-03	TBC-01	1999 Dec	12	7.1	<0.03	263	171	<5	0	0	20.06.01
DWH-16	Md. Abdur Rahman	Tangbari	UBJ-03	TBC-01	1999 Dec	10	7.4	<0.03	325	211	<5	0	3	20.06.01
DWH-17	Md. Sohorab (Police)	Tangbari	UBJ-03	TBC-01	1999 Dec	13	7.0	<0.03	349	227	<5	0	0	20.06.01
DWH-18	Md. Jahur Ali	Tangbari	UBJ-03	TBC-01	1999 Nov	13	6.9	<0.03	285	185	<5	0	3	20.06.01
DWH-19	Md. Lutfor Rahman	Tangbari	UBJ-03	TBC-01	1999 Nov	15	7.0	<0.03	168	109	<5	0	0	20.06.01
DWH-20	Md. Mojhar Sheikh	Kabaskanda	UBJ-03	TBC-01	1999 Dec	10	7.0	<0.03	365	237	<5	0	0	20.06.01
DWH-21	Md. Sohorab Ali	Kabaskanda	UBJ-03	TBC-01	1999 Dec	15	7.0	<0.03	265	172	<5	0	0	20.06.01
DWH-22	Md. Jalil Sheikh	Kabaskanda	UBJ-03	TBC-01	1999 Nov	10	6.9	<0.03	282	183	<5	0	1	20.06.01
DWH-23	Md. Juran Mondol	Kabaskanda	UBJ-03	TBC-01	1999 Dec	13	6.8	<0.03	320	208	<5	0	_2_	20.06.01
DWH-24	Md. Monnaf	Sinduri	UBJ-03	TBC-01	1999 Nov	12	7.0	<0.03	315	205	<5	0	0	20.06.01
DWH-25	Md. Abdur Rajjak Master	Natiabari	UBJ-03	TBC-01	1999 Dec	15	7.0	<0.03	252	164	<5	0	0	20.06.01
DWH-26	Md. Sahadot Hossain	Natiabari	UBJ-03	TBC-01	1999 Nov	10	7.0	<0.03	346	225	<5	0	0	20.06.01
DWH-27	Md. Rohmot Ali	Rajnarayonpur	UBJ-03	TBC-01	1999 Nov	10	7.2	<0.03	292	190	<5	0	2	20.06.01
DWH-28	Md. Ali Saheb	Rajnarayonpur	UBJ-03	TBC-01	1999 Nov	10	7.0	<0.03	304	198	<5	0	3	20.06.01
DWH-29	Md. Torab Ali	Rajnarayonpur	UBJ-03	TBC-01	1999 Dec	11	7.1	<0.03	269	175	<5	0	1	20.06.01
DWH-30	Md. Sattar Sheikh	Sibpur	UBJ-03	TBC-01	1999 Nov	10	7.3	<0.03	214	139	<5	0	0	20.06.01
DWH-31	Md. Ahmed Ali	Sibpur	UBJ-03	TBC-01	1999 Dec	9	7.2	<0.03	198	129	<5	0	0	20.06.01
DWH-32	Md. Mirza Ruhulmin	Aminpur	UBJ-03	TBC-01	1999 Dec	15	7.0	<0.03	282	183	<5	0	_2_	20.06.01
DWH-33	Md. Harun Uddin	Aminpur	UBJ-03	TBC-01	1999 Dec	12	7.0	<0.03	322	209	<5	0	1	20.06.01
DWH-34	Md. Hosen Sheikh	Aminpur	UBJ-03	TBC-01	1999 Nov	14	7.5	<0.03	187	122	<5	0	1	20.06.01
DWH-35	Imam Mirjapur Jame	Mirzapur	UBJ-03	TBC-01	1999 Nov	12	7.1	<0.03	189	189	<5	0	0	20.06.01
DWH-36	Md. Motaleb Hossain Khas	Aminpur	UBJ-03	TBC-01	1999 Dec	11	7.2	<0.03	340	222	<5	0	2	20.06.01
DWH-37	Md. Chad Ali Kash	Aminpur	UBJ-03	TBC-01	1999 Nov	14	7.0	<0.03	241	157	<5	0	1	20.06.01
DWH-38	Md. Akkas Ali	Aminpur	UBJ-03	TBC-01	1999 Nov	10	7.0	<0.03	342	222	<5	0	0	20.06.01
DWH-39	Md. Sahabuddin Master	Rajnarayonpur	UBJ-03	TBC-01	1999 Nov	10	7.0	<0.03	305	198	<5	0	5	20.06.01
DWH-40	Mukul Malitha	Ruppur (Malithapara	UIP-01	TIC-02	2003 Jan	40	7.5	<0.03	202	131	<5	0	1	25.01.03
DWH-41	Mrs. Salina Khatun	Ruppur (Biswaspara	UIP-01	TIC-02	2003 Jan	37	7.0	<0.03	172	112	<5	0	3	25.01.03
DWH-42	Mrs. Samshunnahar	Ruppur (Charabottala	UIP-01	TIC-02	2003 Jan	53	7.1	<0.03	198	127	<5	0	0	25.01.03
DWH-43	Mrs. Rashida Khatun	Babulchara	UIA-01	TIC-02	2003 Jan	48	7.1	<0.03	211	137	<5	0	1	25.01.03
DWH-44	Md. Harunar Rashid	Durgapur	USA-01	TSC-03	2003 Jan	28	7.4	<0.03	222	144	<5	0	3	25.01.03
DWH-45	Mrs. Samshunnahar	Gopalpur	USK-01	TShC-05	2003 Jan	20	7.0	<0.03	282	183	<5	0	_2_	25.01.03
DWH-46	Md. Abdul Momin Khan	Sayedpur (Dangapara)	USA-01	TSC-03	2003 Jan	35	7.2	<0.03	298	194	<5	0	5	25.01.03
DWH-47	Md. Mojibur Rahman	Ahmedpur	USA-01	TSC-03	2003 Jan	32	7.3	<0.03	301	196	<5	0	0	25.01.03
DWH-48	Md. Khorshed Alam	Koromja	UBK-01	TBC-01	2003 Jan	28	6.9	<0.03	314	204	<5	0	1	25.01.03
DWH-49	Md. Mamunur Rashid	Sayedpur (Ujanpur)	USA-01	TSC-03	2003 Jan	32	7.1	<0.03	382	248	<5	0	3	25.01.03
DWH-50	Md. Zinna	Ahmedpur	USA-01	TSC-03	2003 Jan	30	7.4	<0.03	203	132	<5	0	4	25.01.03
DWH-51	Md. Azim Uddin	Bhabanipur	UBR-01	TBC-01	2003 Jan	28	7.4	<0.03	168	109	<5	0	1	25.01.03
DWH-52	Md. Badsha Master	Durgapur	USA-01	TSC-03	2003 Jan	28	7.0	<0.03	321	209	<5	0	_2_	25.01.03
DWH-53	Sree Nironjan Kumar	Sagorkandi	UBM-01	TBC-01	2003 Jan	35	7.1	<0.03	354	230	<5	0	0	25.01.03
DWH-54	Md. Raton Chowdhuri	Arifpur	UPM-01	TPC-04	2003 Jan	15	7.3	<0.03	265	172	<5	0	7	25.01.03
DWH-55	Md. Abdul Awal	Bhabanipur	UBR-02	TBC-01	2003 Jan	27	7.0	<0.03	312	203	<5	0	5	25.01.03
DWH-56	Sree Sudha Ranjan	Shagorkandi	UBM-01	TBC-01	2003 Jan	26	7.2	<0.03	255	166	<5	0	8	25.01.03
DWH-57	Md. Rabiul Islam	Char Ruppur	UIP-01	TIC-02	2004 Jan	24	7.3	<0.03	265	172	<5	0	_2_	28.01.04
DWH-58	Md. Zahidul Haq	Char Ruppur	UIP-01	TIC-02	2004 Jan	25	7.0	<0.03	346	225	<5	0	1	28.01.04
DWH-59	Md. Shahjahan	Char Ruppur	UIP-01	TIC-02	2004 Jan	25	7.3	<0.03	269	175	<5	0	4	28.01.04
DWH-60	Md. Iddris Ali	Arippur	UPM-04	TPC-04	2004 Jan	39	7.0	<0.03	187	122	<5	0	_2_	28.01.04
DWH-61	Md. Shamim Hossain	Arippur	UPM-04	TPC-04	2004 Jan	27	7.4	<0.03	340	221	<5	0	1	28.01.04
DWH-62	Md. Mizanur Rahman	Bishawnathpur	UBJ-03	TBC-01	2004 Jan	27	6.9	<0.03	241	157	<5	0	3	28.01.04
DWH-63	Mrs. Shahanara Lipi	Dariapur	USA-01	TSC-03	2004 Jan	25	7.1	<0.03	305	198	<5	0	4	28.01.04
DWH-64	Md. Abul Raja	Birahimpur	USA-01	TSC-03	2004 Jan	28	7.3	<0.03	202	131	<5	0	0	28.01.04
DWH-65	Md. Habibur Rahman	Rajnaraynpur	UBJ-03	TBC-01	2004 Jan	24	7.2	<0.03	211	137	<5	0	0	28.01.04
DWH-66	Md. Raij Shikder	Bhuya Para	UBR-02	TBC-01	2004 Jan	25	7.2	<0.03	282	183	<5	0	5	28.01.04

More complete measurements are available at: http://phys4.harvard.edu/∼wilson/remediation/dugwells/DCH_Phase1_Bacteria_tests.xls or http://DCHtests.arsenic.ws

As=Arsenic;

Conc.=Concentration;

Cond.=Conductivity;

DW=Dugwell;

NTU=Nephelometric Turbidity Unit;

TBC=Thana Bera Code;

TIC=Thana Ishwardi Code;

TDS=Total dissolved solid;

Turb=Turbidity;

UBJ=Upazila Bera Jatshakhini;

UBM=Upazila Bera Masumdia;

UBR=Upazila Bera Ruppur;

UIA=Upazila Ishwardi Awtapara;

UIP=Upazila Ishwardi Pakshi;

WHO=World Health Organization
